# Improvement capability and performance: a qualitative study of maternity services providers in the UK

**DOI:** 10.1093/intqhc/mzy081

**Published:** 2018-04-16

**Authors:** Sarah Darley, Kieran Walshe, Ruth Boaden, Nathan Proudlove, Mhorag Goff

**Affiliations:** 1 Centre for Primary Care, School of Health Sciences, Oxford Road, Manchester, UK; 2 Health Management Group, Alliance Manchester Business School, University of Manchester, Manchester, UK; 3 NIHR Collaboration for Leadership in Applied Health Research and Care (CLAHRC), Alliance Manchester Business School, Booth Street West, Manchester, UK; 4 Health Services Research Centre, Alliance Manchester Business School, University of Manchester, Manchester, UK

**Keywords:** quality improvement, improvement capability, maternity services

## Abstract

**Objective:**

We explore variations in service performance and quality improvement across healthcare organisations using the concept of improvement capability. We draw upon a theoretically informed framework comprising eight dimensions of improvement capability, firstly to describe and compare quality improvement within healthcare organisations and, secondly to investigate the interactions between organisational performance and improvement capability.

**Design:**

A multiple qualitative case study using semi-structured interviews guided by the improvement capability framework.

**Setting:**

Five National Health Service maternity services sites across the UK. We focused on maternity services due to high levels of variation in quality and the availability of performance metrics which enabled us to select organisations from across the performance spectrum.

**Participants:**

About 52 hospital staff members across the five case studies in positions relevant to the research questions, including midwives, obstetricians and clinical managers/leaders.

**Main Outcome Measure:**

A qualitative analysis of narratives of quality improvement and performance in the five case studies, using the improvement capability framework as an analytic device to compare and contrast cases.

**Results:**

The improvement capability framework has utility in analysing quality improvement within and across organisations. Qualitative differences in the configurations of improvement capability were identified across all providers but were particularly striking between higher and lower performing organisations.

**Conclusions:**

The improvement capability framework is a useful tool for healthcare organisations to assess, manage and develop their own improvement capabilities. We identified an interaction between performance and improvement capability; higher performing organisations appeared to have more developed improvement capabilities, though the meaning of this relationship requires further research.

## Introduction

The highly variable progress and impact of quality improvement programmes in healthcare organisations [1] is often attributed to two factors: the improvement intervention itself and the fidelity with which it is implemented [2]; and the nature, history, trajectory and other attributes of the organisation in which it is implemented, often labelled organisational context [3, 4]. There have been a number of approaches and attempts to provide guidance on how to describe improvement interventions and their evaluation [5] to describe the salient features of organisational context [6], and to advocate for research and implementation to take greater account of such contextual factors [7]. The division of intervention and context is arguably somewhat artificial because the two interact in multiple, complex and dynamic ways [8]. Descriptions of contextual factors or attributes without a rationale founded in relevant organisational theory seem unlikely to produce much explanatory insight [9].

However, there is a substantial relevant literature, in healthcare and other sectors, on what has been termed improvement capability, defined for the purposes of this paper as ‘the organisational ability to intentionally and systematically use improvement approaches, methods and practices, to change processes and products/services to generate improved performance’ [10] (p. 5). This definition comes from a recent integrative review of improvement capability by Furnival *et al.* which highlighted the absence of empirically tested and validated frameworks for assessing improvement capability and identified a number of competing definitions and 70 different assessment tools or frameworks with somewhat divergent underlying theoretical models, constructs and measurement items. The review produced a synthesis of those constructs into a framework of eight core dimensions of improvement capability: service-user focus, stakeholder focus, organisational culture, employee commitment, leadership commitment, data and performance, process improvement and learning, and strategy and governance. Importantly, it also located the ideas and concepts of improvement capability in the much wider literature on organisational resources and capabilities and their relationships with organisational performance. This ‘dynamic capabilities view’ [11–14] has been widely used and empirically tested [15–18]; though it is not without its critics who highlight a lack of consistency in definitions [19, 20], a tautological tendency [21] and an absence of approaches to assessment [22].

We had two main aims in this study. First, we wanted to test out Furnival *et al.*’s improvement capability framework for the first time empirically, to see whether it was helpful in describing and explaining the development and progress of quality improvement programmes in healthcare organisations, and to see whether we could define the eight dimensions more fully through empirical study. Second, we wanted to explore the interactions between organisational performance and improvement capability, by seeing whether organisations with differing performance on available metrics had different configurations of improvement capability (by which we mean the qualitative description of their capabilities when mapped to the eight dimensions of the improvement capability framework). We used the improvement capability framework to guide our data generation and analysis, which are outlined in the following section.

## Methods

### Setting and sample

We undertook the study in maternity services in the UK because of the availability of a wide range of quantitative performance metrics, the known existence of variations in quality and safety and the high salience of this service area resulting from past high-profile failures in care [23, 24]. In addition, because we do not know how much improvement capability might vary across or within healthcare organisations by specialty or clinical service area, we chose maternity services because they tend to be relatively self-contained and less co-dependent on other specialties than many others in acute care.

We selected case studies of National Health Service (NHS) maternity service providers in the UK to represent a range of performance on publicly available quantitative metrics drawing on routine data based on hospital admissions, national patient surveys and regulatory reviews [25–29]. We assembled a set of 38 indicators covering four domains of quality: clinical care, mortality, regulatory assessment and patient experience*.* Monte Carlo simulation techniques [30] were then used to model the results of aggregating the indicators using many different relative weightings and we then calculated how often each provider appeared in the top or bottom decile of the performance distribution.

This method identified eight providers which appeared in the top decile of performance on more than 50% of occasions and eleven which appeared in the bottom decile of performance more than 50% of occasions, with the remaining providers being ‘middling’ performers. We selected five maternity service providers for our case studies based on this quantitative assessment, contextual factors (geography, neonatal care level, size of unit measured in number of deliveries per annum), and willingness to participate. Our cases are described in Table 1.

**Table 1:** Case study sites

Case study	Narrative description	Performance
Trust A	A Foundation Trust with ~4500 annual births and an LNU; progressed from being placed in special measures a few years ago to currently holding an ‘Outstanding’ CQC rating for the maternity services department. The organisation had a high level of engagement with external organisations, such as universities, the CCG, the Allied Health Science Network, NICE and RCOG. A blended approach was taken to improvement methods, which included PDSA and lean methods and aimed to provide a toolkit of improvement as part of their improvement academy.	High (92% of the time in the top decile, never in the bottom decile)
Trust B	A Foundation Trust with ~7750 annual births and a SCU and NICU. The maternity services department currently has a ‘Good’ CQC rating. The organisation had a high level of engagement with some external organisations, such as universities, the regional strategic clinical network and the Allied Health Science Network, No specific methodologies were identified as being followed for improvement.	Middle (1% of the time in top decile,0% of the time in bottom decile)
Trust C	A Foundation Trust with ~6 000 annual births and a NICU; placed in financial special measures last year and the maternity services department is currently rated as ‘Requires Improvement’ by CQC. The organisation had few links to external organisations and networks. No specific methodologies were identified as being followed for improvement.	Middle (17% of the time in top decile,1% of the time in bottom decile)
Trust D	A Foundation Trust with ~8 000 annual births across two hospitals and a SCU and LNU; contains two recently merged sites, one of which was a poor performer before the merger. The maternity services department was currently rated by the CQC as ‘Good’. Strong networks exist with other maternity services in the area and there are partnerships with external organisations to learn about approaches to quality. There was a planned systematic approach to quality improvement with relevant training opportunities.	Middle (0% of the time in top decile,9% of the time in bottom decile)
Trust E	A Foundation Trust with ~3 000 annual births and an LNU; the maternity services department was investigated for a series of perinatal deaths prior to the most recent rating of ‘Requires Improvement’ by CQC. External standards and pressure from stakeholders had contributed to quality improvements and the drive for improvement seemed to come externally rather than internally. Bodies such as the CQC and RCOG are seen as being needed to bring about change where there is a perceived lack of Trust level support. There was no systematic approach to quality improvement	Low (75% of the time in the bottom decile, never in the top decile)

*Note*: LNU, Local Neonatal Unit; SCU, Special Care Unit; NICU, Neonatal Intensive Care Unit.

As Table 1 outlines, there were a wide range of improvement methods used across the five organisations and all had experienced regulatory inspections though with differing outcomes. The organisations had varying levels of external engagement and some differences in their internal management structures.

### Instruments and procedures

We undertook semi-structured interviews across the five case studies with 52 hospital staff members in positions relevant to the research questions (e.g. midwives, obstetricians, quality/audit leads, governance and service directors and clinical managers/leaders). Participants were selected to include a range of perspectives and experiences from different levels in each organisation. All interviews followed the same interview schedule, which aimed to explore how improvement happens and to understand any aspects that help or hinder improvement within each organisation. The interview questions were guided by the eight dimensions of the improvement capability framework, and structured questions enabled comparisons to be made across the different cases [31]. Relevant documents, such as action plans, reports and meeting minutes were also reviewed to enhance the understanding of each case [32]. Ethical approval for the research was granted by the University of Manchester and coordinated research governance approval was given by the Health Research Authority (ref: 184263) and the participating NHS organisations. In this paper the case study sites and research participants are anonymised.

Interviews lasted between 30 min and 1 h. Interviews were recorded with the permission of each participant and transcribed verbatim. To address potential informant bias, triangulation of data was achieved by including a range of participants in the study and through the review of documents.

### Data analysis

Analysis involved deductive coding of interview transcripts using the improvement capability framework, which was then expanded from the data inductively. This deductive–inductive cycle not only allowed the data to be interpreted through the improvement capability framework, but also enabled the development and expansion of the framework from the data through various subthemes. Cross case analysis was then conducted to interpret whether improvement capability appeared to be configured differently across the differently performing organisations. The software Nvivo was used as a tool to manage and analyse data and all data generated within the study was stored in Nvivo, including audio files, documents and transcripts. These practices helped to avoid data overload and manage the large amount of data commonly collected in case studies [33]. Furthermore, Nvivo provided a useful place to keep an audit trail to detail and justify data collection [34] and support the sincerity and transparency of the research [35]. This audit trail included, for example, all generated data, notes and reflections following data generation and analysis.

## Results

We present the findings from the study below. First, we explore whether the eight dimensions of improvement capability derived from the review are found in our qualitative data, and report on the results of inductive coding within each dimension. Second, we compare and contrast the configurations of improvement capability found in our high and low performing providers (Trust A and Trust E). Third, we examine in detail the configurations of improvement capability in two example dimensions—leadership commitment and process improvement and learning—across all case study sites (space precludes such an analysis in this paper for all eight dimensions).

### Identifying and describing the dimensions of improvement capability

The improvement capability dimensions [10] were clearly identifiable in all the cases as is illustrated in Table 2, which lists each dimension, the subthemes identified in our analysis, and a typical example from our interview data. The subthemes that emerged from inductive analysis provide a more detailed and nuanced understanding of each dimension and enabled us to ‘follow’ rather than ‘lead’ the data [32]. For example, we found that leadership commitment within an organisation covers a wide area and the subthemes that form this dimension help to highlight particular aspects of leadership that affect on improvement, such as the level of support demonstrated by leaders for improvement and leaders’ focus on improvement as perceived by staff.

**Table 2:** Improvement capability dimensions

Improvement capability dimension [10]	Subthemes from case study data	Example from case study data
*Service-user focus*: Identification and meeting of current and emergent service needs and expectations of service users.	Feedback Needs Involvement Safety Choice Experience Centre	‘I think if you bring it back to the women, then [staff] can see it because it’s not about you doing what I’m telling you, it’s for this woman’s experience.’ (Head of Midwifery, Trust D)
*Stakeholders and supplier focus***:** The role of external organisations and regulatory bodies in improvement.	Engagement Standards Contribution	‘I think we are driven by national guidance from the NICE guidelines and you know, providing evidence based information and guidelines and policies and trying to improve the standards by working with the most current evidence based practice really.’ (Ward Manager, Trust B)
*Organisational culture*: Core values, attitudes and norms; underlying ideologies and assumptions.	Context Innovation Openness Values Reputation Relationships	‘[T]here’s a better listening culture now as well I feel. Whether that’s due to personalities or just because of recognising we had a bit of a heavy blame culture.’ (Matron, Trust E)
*Employee commitment*: Motivation of employees and their support for improvement.	Autonomy Role Voice Motivation Resources Involvement	‘I do find this organisation quite disempowering. There’s a lot of people with a lot of really good ideas, want to take things forward, but you just get obstacles in the way on quite a few things that you want to change.’ (Midwife, Information Technology specialist, Trust C)
*Leadership commitment*: Support for improvement demonstrated by organisational leaders.	Visibility Attitude Accountability Focus Communication Support Skills	‘[The CEO] is very visible and I think that’s a really good role model. So, you know, for example, when he was coming to visit one day I was telling people, the chief exec’s coming today, they were like, oh yeah, that’s okay. He knows me. And he did. He was like, hi - knows the ward clerk by name, hello, smiles to everybody.’ (Service Line Lead for Maternity, Trust D)
*Data and performance*: Use of data and analysis methods to support improvement activity.	Clinical measures Data accessibility Data quality Data use Technical systems	‘I think there is a culture of accepting all the data, good or bad and reacting to it. I think there’s almost a knowledge that we have to immediately react to it in some way or another. It can’t just be left.’ (Consultant Obstetrician, Trust A)
*Process improvement and learning: *Systematic methods and ongoing processes to make improvements.	Ongoing practice Training Sharing Supervision Methods	‘There are processes, but I don’t know if they’re dictated by the Trust or if they’re just what people have learned and what they’ve seen.’ (Midwife, Quality Improvement specialist, Trust B)
*Strategy and governance*: Implementation and management of organisational aims and objectives.	Goals Plans Specific to maternity Staff structure	‘[I]t’s all very well having all these missives coming from above - this is what needs to happen - but you need the right people in place who can produce that change.’ (Clinical Governance and Audit Lead, Trust E)

### Differences in improvement capability between high and low performing providers

Qualitative differences in the configurations of improvement capability were identified across the case studies but they were most clearly identified in the two cases at each end of the performance range: Trust E, a ‘low’ performer, and Trust A, a ‘high’ performer (see Table 3).

**Table 3:** Contrasting cases of improvement capability in high and low performing providers

Improvement capability dimension	Trust E	Trust A
Service-user focus	‘[W]e will participate in the national reports and surveys, but we don’t generate our own annual surveys or anything like that.’ (Governance Manager)	‘[W]e take on board, as well, any feedback from surveys, so I regularly run surveys on the wards.’ (Divisional Audit and Research Midwife)
Stakeholder and supplier focus	‘[M]aternity networks, I’ve been to one or two but I can’t say I’ve had the time or exposure or the opportunity really.’ (Matron)	‘[B]y networking and sitting on other groups you tend to be ahead of the game with some of the things that are happening.’ (Divisional Audit and Research Midwife)
Organisational culture	‘[T]he culture here was historically a bit of a downtrodden nursing workforce and a pretty outspoken consultant body in part and a pretty complacent consultant body in other parts.’ (Director of Nursing)	‘[W]e’ve always first of all had a multidisciplinary approach, midwives and doctors working together’ (Consultant Obstetrician Gynaecologist)
Employee commitment	‘[S]taff were demoralised - we were wanting to implement things, they didn’t want to try.’ (Governance Manager)	‘[P]eople are coming forward with ideas, people are putting themselves forward for being involved in an improvement, people are enroling in things.’ (Clinical Lead for Improvement)
Leadership commitment	‘[W]e’re going through a lot of change now, I have no idea what the reporting structure is at the moment, we’re in a bit of a limbo.’ (Clinical Governance and Audit Lead)	‘[W]e have very robust governance… we can’t just verbalise - this is what we do - and we have to be able to prove this is what we are doing.’ (Head of Midwifery)
Data and performance	‘[A]t the moment we have no data, electronic data, they’re unable to run any reports.’ (Clinical Governance and Audit Lead)	‘[W]e have very good information technology systems that help us to extract data.’ (Divisional Audit and Research Midwife)
Process improvement and learning	‘[W]e don’t have, this is our kitbag of quality improvement tools, our preference is lean methodology and PDSA, this is how we will identify projects for improvement, so we’ve no strict methodology for that.’ (Director of Nursing)	‘[M]y aim is to give people a toolkit of improvement where they say, oh this applies here, I’ll go and do this.’ (Improvement Training Lead)
Strategy and governance	‘I think it’s very much driven by issues we identify along the way rather than having a planned strategy.’ (Clinical Governance and Audit Lead)	‘[E]ach year or each certain period of time we would actually decide on what our priorities are or get everyone individually in their practice to decide what their own priority is and then turn it into a formalised project and face it seriously as a team.’ (Consultant Obstetrician)

As can be seen in Table 3, Trusts A and E are starkly different not only in their performance according to the quality metrics but also in their configurations of improvement capability. For example, whereas improvement activities in Trust E were described by the Clinical Governance and Audit Lead as being ‘driven towards pleasing external bodies’, in Trust A the Supervisor of Midwives described an active focus on service users as being ‘a great driver for any quality improvement’. Similarly, staff in Trust A reported feeling able and ‘empowered’ to initiate improvement and encouraged not just to highlight problems but to also come up with ideas and solutions. Such encouragement was demonstrated through the annual awards for improvement projects and the development of ‘improvement champions’ to motivate and guide other staff members.

In Trust E, staff described improvement activities as being led by a limited number of proactive individuals rather than collectively as part of directorate culture. There appeared to be no systematic approach to quality improvement and future goals and priorities were described as being driven by ongoing issues, rather than following any ‘planned strategy’. In contrast, future goals and priorities in Trust A were collaboratively planned in advance and structural changes had been made across the Trust with the aim of improvement. In particular, much effort had gone into developing and teaching systematic approaches to quality improvement and staff described feeling empowered with a ‘toolkit of improvement’ that gave them ‘ownership’ of improvement activity.

In short, the large differences in performance according to available quality metrics between Trust A (in the top decile 92% of the time) and Trust E (in the bottom decile 75% of the time) were matched by equally marked differences in their configurations of improvement capability.

### Differences in improvement capability across the range of performance

Although the three ‘middling’ performing cases were not easily distinguishable in the quantitative analysis of performance metrics, we found there were qualitative differences in improvement capability as this section will outline. Broadly, Trust D appeared to be similar in many ways to Trust A (our high performer) in terms of their configuration of improvement capability, whereas Trusts B and C seemed to share some similarities with Trust E, our low performer. Below, we focus on two of the eight improvement capability dimensions (leadership commitment and process improvement and learning), using the subthemes within these dimensions to explore the similarities and differences between the cases in further detail. All eight dimensions could not be discussed in detail within the scope of this paper, but we believe that the two dimensions presented are reflective of findings from the other dimensions.

### Leadership commitment

The improvement capability dimension of leadership commitment concerns the support for improvement demonstrated by organisational leaders, which in our study included both trust and divisional leaders. This section presents how the subthemes within this dimension appeared across the cases (summarised in Table 4) and concentrates on differences regarding the support and focus of leaders for improvement activity, and how this is communicated to staff, including the visibility of senior leaders and existing accountability structures.

**Table 4:** Leadership commitment

Performance	Low	Middling	Middling	Middling	High
**Subthemes**	**Trust E**	**Trust C**	**Trust B**	**Trust D**	**Trust A**
*Support*	Perceived lack of support for and interest in maternity from trust leaders until an emergency.	Perceived lack of support for and interest in maternity from trust leaders until an emergency.	Perceived lack of support for and interest in maternity from divisional leaders.	Perceived support for and interest in maternity from trust leaders.	Perceived support for and interest in maternity from trust leaders.
*Focus*	Perceived commitment to and valuing of improvement from some trust leaders.	Perceived lack of commitment to and valuing of improvement from trust leaders.	Perceived commitment to and valuing of improvement from trust leaders.	Perceived commitment to and valuing of improvement from trust leaders.	Perceived commitment to and valuing of improvement from trust leaders.
*Communication*	Multiple communication channels downwards.	Multiple communication channels downwards.	Perceived lack of effective communications.	Multiple communication channels.	Multiple communication channels upwards and downwards.
*Accountability*	Perceived lack of reporting and accountability structures.	Perceived lack of reporting and accountability structures.	Clear reporting and accountability structures for clinical managers.	Clear reporting and accountability structures to the trust.	Clear reporting and accountability structures to the trust.
*Skills*	Leadership training is available but there is limited time for staff to put skills into practice.	Lack of leadership training is perceived to result in a lack of focus on quality.	Perceived emphasis on leadership training for clinicians.	Leadership training is regularly available to a wide range of staff.	Comprehensive leadership training aims to develop leaders internally.
*Visibility*	Divisional leaders are perceived as visible to staff in management positions but disconnected from front-line staff.	Trust leaders are perceived as disconnected from divisional staff, but the Head of Midwifery (HOM) is perceived as an important link.	Divisional leaders are perceived as disconnected, except the HOM.	Divisional leaders are perceived by staff as visible.	Trust and divisional leaders are perceived by staff as visible and transparent.
*Attitude*	Trust leaders are perceived by staff as approachable, but there were previous concerns of bullying at divisional level.	Previous concerns of bullying at trust level, but the HOM is perceived as proactively addressing and changing this culture.	Trust leaders are perceived by staff as approachable.	Trust leaders are perceived as caring and approachable.	Trust leaders are perceived by staff as open and approachable.

In Trusts A and D staff perceived strong support for improvement from organisational and divisional leaders which appeared to have a motivational effect. However, in Trusts B, C and E staff expressed feeling a lack of support and interest from leaders, except when something in the service ‘goes wrong’ or fails:

‘We’re not really heard or seen unless it goes wrong. When it goes wrong then I’m upstairs talking to the Chief Executive or the Medical Director or the Director of Nursing - what the hell’s going on? - but the rest of the time I don’t think they’re that interested in us to be honest really.’ (Clinical Director, Obstetrics, Trust C)

The perceived focus of leaders and their values, vision and priorities also impacted on improvement activities. In Trusts A and D leaders were described by staff as having ‘a drive towards quality improvement projects’ and a focus on quality rather than finance. In contrast, in Trusts C and E finance was perceived as being more of a priority for leaders than patient care:

‘They do make a lot of noise about it, I think they are quite keen on improvement and delivering change, as long as it’s cost neutral and I think that’s the problem really - they want all these ideas, they want us to change things, but then when there’s money involved then it’s, like, you know, there’s no funding.’ (Obstetrician and Governance lead, Trust C)

‘[T]hat’s their main priority, financial viability because quality costs. And I can understand their point of view, but you can’t let quality drop.’ (Clinical Lead and Consultant Obstetrician and Gynaecologist, Trust E)

The focus of leaders on improvement was communicated in different ways across the cases and the extent to which staff felt able to contribute to this focus also differed. In Trusts A and D communications from and with leaders were described as multidirectional:

‘[I]t’s so important to feedback, because sometimes when you do drain extra resources needed to improve services and people resist it. When you can demonstrate the value and they can see the improvement in patient care and outcomes that makes a difference. But we also feed these up at ward meetings, to the forums, to then the governance, to risk and compliance and these also get escalated up to trust board as well. So it’s about filtering up to filtering it down.’ (Midwife, Audit and Research specialist, Trust A)

Staff in Trusts B, C and E expressed a feeling of disconnection from senior leaders created by a one way, downward flow of communication and lack of visible presence:

‘I do feel, personally, that there is above the Head of Midwifery there is obviously a group of people that have all these meetings. But I don’t always feel that all of that comes down to us.’ (Ward Manager, Trust B)

‘[W]e’re always asked to escalate something and we don’t know what happens to it once it’s been escalated… Sometimes it can be a bit frustrating, particularly for people who are on the shop floor when we are supposed to be the link between them and management. So I think there can be a little bit of disconnect from senior management because they don’t disseminate in the right channels.’ (Obstetrician and Governance Lead, Trust C)

‘I would say that we never had a direct access to anybody above the head of midwifery really… I feel that it wasn’t a two way street. So they raised their concerns and gave us their objectives, and they got fed down to us via the head of midwifery and matrons, but it almost felt punitive - if you don’t do this, if you don’t do that. I feel that we weren’t afforded the same voice the other way.’ (Labour Ward Manager, Trust E)

Improvement appeared to be enabled in Trusts A and D through strong support from senior leaders, which was communicated in various ways with opportunities for staff to contribute. These opportunities were rather more lacking in Trusts B, C and E, where staff often felt disconnected from and unimportant to senior leaders.

### Process improvement and learning

The improvement capability dimension of process improvement and learning concerns the availability and use of systematic methods and ongoing processes to enable improvement. This section presents how the subthemes within this dimension appeared across the cases (summarised in Table 5) and concentrates on differences regarding methods used for improvement, how learning around these methods is shared and how improvement activity is supported by training and supervision.

**Table 5:** Process improvement and learning

Performance	Low	Middling	Middling	Middling	High
**Subthemes**	**Trust E**	**Trust C**	**Trust B**	**Trust D**	**Trust A**
*Ongoing practice*	Emphasis on learning from mistakes.	Perceived lack of opportunities to reflect and learn from mistakes.	Emphasis on learning from mistakes, reflection and embedding learning into practice.	Emphasis on reflection, learning from mistakes and embedding learning into practice.	Emphasis on reflection and learning from mistakes.
*Training*	Limited available improvement training.	Range of improvement training available but limited resources for staff engagement.	Range of improvement training available but limited time for staff engagement.	Range of improvement training available with an emphasis on reflective practice.	Range of improvement training available with an emphasis and embedding learning into practice.
*Sharing*	Structures enable internal sharing of learning. A perceived lack of learning and sharing externally.	Lack of time prevents staff coming together to share learning. A perceived lack of learning and sharing externally.	Sharing learning across the organisation is difficult due to its size. Links with external organisations and networks enable sharing of information and learning.	Structures in place to enable internal sharing of learning. Links with external organisations and networks enable sharing of information and learning.	Structures enable internal sharing of learning. Links with external organisations and networks enable sharing of information and learning.
*Supervision*	No mention of appraisals or supervision.	No structured supervision and ineffective appraisals.	Staff supervision and appraisals provide opportunities for development and feedback.	Staff supervision and appraisals provide opportunities for development and feedback.	Staff supervision and appraisals provide opportunities for development and feedback.
*Methods*	No systematic or planned approach to quality improvement.	No systematic or planned approach to quality improvement.	No systematic or planned approach to quality improvement.	Systematic and planned approach to quality improvement.	Systematic and planned approach to quality improvement.

Methods and tools drawn upon for improvement varied across trusts, from Trust E where staff described a strong reliance on audits to Trust A where a more flexible project-based approach had been developed:

‘[M]y aim is to give people a toolkit of improvement where they say, this applies here, I’ll go and do this. I don’t have a kind of one size fits all approach and also if I’m honest I don’t really mind what tool you use, as long as it gives you what you need it to give you.’ (Improvement Training Lead, Trust A)

Staff in Trust A described a systematic approach to quality improvement, going beyond a reliance on audit, which was described as a way to identify a problem but not prescribing how to solve it. A systematic approach was described as lacking in Trusts E and C, which was viewed as a barrier to improvement activity. Staff in Trust B highlighted the use of methodologies such as the PDSA cycle, but also described a similar lack of a systematic approach to improvement:

‘There are processes, but I don’t know if they’re dictated by the Trust or if they’re just what people have learned and what they’ve seen.’ (Midwife, Quality and Audit specialist, Trust B)

All trusts except Trust E provided training in improvement methods, however staff in Trusts C and B reported that limited resources, in particular staff time, prevented them from participating in such learning opportunities. Time to put ‘training into practice’ and opportunities to share learning were valued by staff across the cases:

‘But, it wasn’t a line in a risk newsletter or an email. It was proper time out and intervals of sharing - this is what I learnt and this has changed a little bit.’ (Midwife, Trust D)

A more ‘organic’, ongoing ‘learning on the job’ process was described by staff in Trust E, which made monitoring and assessing staff development difficult. In Trusts A and D multidisciplinary training and development provided opportunities for service improvement, which was supported through supervision and appraisals:

‘[W]e need to encourage our staff to develop themselves, so by doing that and by doing projects they not only develop themselves, they do service improvement.’ (Matron, Trust A)

Staff in both Trusts A and D placed much importance on sharing learning and knowledge internally, through various communication strategies including multidisciplinary meetings. Staff described the recent merger in Trust D as providing a particularly useful opportunity to share learning and ‘best practice’ across both hospital sites:

‘Everybody had a mirror in the other organisation, and I think from that I witnessed quite a number of sharings of, this is my role, that was yours. Almost like a benchmarking going on of, we did this, you did that, and actually this is where we know where we should be and where everybody was, and actually how they were going to get there.’ (Midwife, Trust D)

Staff in Trust B had opportunities to share learning through multiple communication channels and meetings, but said that barriers arose due to the size of the organisation. Staff in Trusts C and E described a more insular environment with limited networks and barriers to sharing learning both externally and internally:

‘I think the area we need to improve on, which we are now working on - is integration with other hospitals, and sharing and learning from other hospitals.’ (Midwifery Lead for Clinical Risk and Risk Manager for Maternity, Trust C)

‘[W]here I’d want to improve would be - we talk about learning from mistakes and I can show you the actual factual proof to show that we’ve discussed it, we’ve sent people the feedback. But actually if I went and said to somebody, did you hear about the mistake that happened on such place, do I think that everybody would know that? Probably not.’ (Director of Nursing, Trust E)

Improvement was enabled in Trusts A and D through a planned systematic approach, along with training and opportunities to share and put learning into practice.

## Discussion

We found the concept of improvement capability and the framework of eight dimensions useful in describing and analysing the organisational capabilities of the five maternity service providers in our case studies, and in seeking more than a superficial description of differences in organisational context and making a more theoretically led analysis. We found the subthemes identified inductively from our qualitative data helpful in moving beyond broad descriptions of improvement capability dimensions like leadership commitment to conceptualise and articulate their meaning in more detail. We have been cautious about describing particular configurations of improvement capability in scalar terms (such as strong and weak, or good and poor) but it seems clear that Trusts A and D in our research had a more developed or mature improvement capability than trusts B, C and E.

Our study provides some empirical evidence that different configurations of improvement capability are associated with differences in organisational performance, a finding which is consistent with prior research on dynamic capabilities [14, 18]. However, the nature of any relationship remains unclear. It seems plausible that organisations with more developed or mature improvement capabilities are more able to improve and sustain performance and therefore more likely to either be high performing or to become high performing. Equally, it is probable that organisations which seem to lack improvement capabilities will not be able, on their own, to bring about performance improvements and may need external support, and there is a risk that when such external support is withdrawn improvements may not be sustained unless their own improvement capabilities have been developed.

We think the main value of the improvement capability framework may be in helping those engaged in quality improvement in healthcare organisations to conceptualise, articulate and self-assess their organisations’ improvement capabilities in order to better understand how to increase the progress and impact of improvement programmes. The framework could also be useful to external bodies such as regulators or improvement agencies who need to decide where to target their limited resources, and to make assessments about both the current performance and future likely trajectory of organisations, but whoever uses it, we think its main value lies more in formative and developmental assessments of improvement capability, rather than in summative judgments.

We recognise the limitations of our study, which was undertaken in one clinical service area, in a small number of case studies, at one point in time and only included research participants from within rather than external to each organisation. There are a number of areas for further research. We need a better understanding of what we have termed configurations of improvement capability: how the different dimensions in the framework are connected or interrelated. We know little about how improvement capability varies within organisations (e.g. between clinical service areas or specialties), or how it changes over time, and longitudinal research could explore these issues and help provide a better understanding of the associations between improvement capability and performance. If we think an organisation’s stock of improvement capabilities is a dynamic resource that requires ongoing work to replenish and maintain, we would surely want to know more about the ways that organisations can develop improvement capabilities, and the interventions they might use. The development of diagnostic tools for assessing or measuring improvement capability could be pursued, though we are cautious about how quantifiable these essentially qualitative attributes may be, and aware of the risks that attempts at measurement and quantification could create perverse incentives and behaviours [36, 37].

## Conclusion

This paper has presented an empirical study of improvement capability and its relationship with organisational performance, which suggests that the concept of improvement capability is useful for understanding quality improvement and improving and sustaining the performance of healthcare organisations.
